# Prevalence of different virulence factors and their association with antimicrobial resistance among *Pseudomonas aeruginosa* clinical isolates from Egypt

**DOI:** 10.1186/s12866-023-02897-8

**Published:** 2023-06-03

**Authors:** Eva A. Edward, Marwa R. El Shehawy, Alaa Abouelfetouh, Elsayed Aboulmagd

**Affiliations:** 1 Department of Microbiology and Immunology, Faculty of Pharmacy, Alexandria University, Alexandria, Egypt; 2 Department of Microbiology and Immunology, Faculty of Pharmacy, Alamein International University, Alamein, Egypt; 3 College of Pharmacy, Arab Academy for Science, Technology and Maritime, Alamein Branch, Alamein, Egypt

**Keywords:** Multi-drug resistance, Virulence genes, Ambroxol, Biofilm inhibition, Alginate, Quantitative reverse transcriptase polymerase chain reaction

## Abstract

**Background:**

Emergence of multi-drug resistant *Pseudomonas aeruginosa,* coupled with the pathogen’s versatile virulence factors, lead to high morbidity and mortality rates. The current study investigated the potential association between the antibiotic resistance and the production of virulence factors among *P. aeruginosa* clinical isolates collected from Alexandria Main University Hospital in Egypt. We also evaluated the potential of the phenotypic detection of virulence factors to reflect virulence as detected by virulence genes presence. The role of alginate in the formation of biofilms and the effect of ambroxol, a mucolytic agent, on the inhibition of biofilm formation were investigated.

**Results:**

A multi-drug resistant phenotype was detected among 79.8% of the isolates. The most predominant virulence factor was biofilm formation (89.4%), while DNase was least detected (10.6%). Pigment production was significantly associated with ceftazidime susceptibility, phospholipase C production was significantly linked to sensitivity to cefepime, and DNase production was significantly associated with intermediate resistance to meropenem. Among the tested virulence genes, *lasB* and *algD* showed the highest prevalence rates (93.3% and 91.3%, respectively), while *toxA* and *plcN* were the least detected ones (46.2% and 53.8%, respectively). Significant association of *toxA* with ceftazidime susceptibility, *exoS* with ceftazidime and aztreonam susceptibility, and *plcH* with piperacillin-tazobactam susceptibility was observed. There was a significant correlation between alkaline protease production and the detection of *algD*, *lasB*, *exoS*, *plcH* and *plcN*; pigment production and the presence of *algD*, *lasB*, *toxA* and *exoS*; and gelatinase production and the existence of *lasB*, *exoS* and *plcH*. Ambroxol showed a high anti-biofilm activity (5% to 92%). Quantitative reverse transcriptase polymerase chain reaction showed that alginate was not an essential matrix component in *P. aeruginosa* biofilms.

**Conclusions:**

High virulence coupled with the isolates’ multi-drug resistance to commonly used antimicrobials would increase morbidity and mortality rates among *P. aeruginosa* infections. Ambroxol that displayed anti-biofilm action could be suggested as an alternative treatment option, yet in vivo studies are required to confirm these findings. We recommend active surveillance of antimicrobial resistance and virulence determinant prevalence for better understanding of coregulatory mechanisms.

**Supplementary Information:**

The online version contains supplementary material available at 10.1186/s12866-023-02897-8.

## Background

*Pseudomonas aeruginosa (P. aeruginosa)* is one of the most clinically important Gram-negative bacteria. It is an opportunistic pathogen responsible for about 10 to 20% of nosocomial infections worldwide [1]. It can cause a wide array of dangerous infections, including ventilator-associated pneumonia, blood stream infections, urinary tract infections, soft tissue infections, and systemic infections [2]. *P. aeruginosa* is inherently resistant to various classes of antibiotics and can acquire resistance to nearly all effective antimicrobial agents leading to the development of multi-drug resistant (MDR) strains [3]. This, coupled with the pathogen’s versatile virulence factors, lead to high morbidity and mortality rates [4].

*P. aeruginosa* virulence factors are classified into cell-associated factors, including adhesins and lipopolysaccharide, as well as secreted factors, such as exotoxin A, proteases, exoenzymes, phospholipases C, pyocyanin, alginate, and DNase [5]. Exotoxin A is responsible for the inhibition of protein synthesis by most *P. aeruginosa* clinical isolates [6], while exoenzyme S possesses multiple injurious effects on the host cells causing cell death [7, 8]. Alginate, a viscous polysaccharide*,* is responsible for the mucoid phenotype commonly isolated from cystic fibrosis (CF) patients [9]. Phospholipases C (both hemolytic and non-hemolytic) hydrolyze the lung surfactant proteins [10, 11].

Pyocyanin is a blue pigment linked to tissue injury via generation of reactive oxygen species [12] and induction of neutrophil apoptosis [13]. DNase produced by* P. aeruginosa* is crucial for the decomposition of the extracellular DNA to be utilized as a source of nutrition [14]. Hemolysin, also produced by this pathogen, lyses different cell types and facilitates the spread of the infections [15]. *P. aeruginosa* proteases are involved in the host’s tissue degradation as well as the inactivation of the components of the immune system [16]. In addition, *P. aeruginosa* can form biofilms in acute and chronic infections leading to increased antibiotic resistance and life-threatening nosocomial persistent infections [17, 18].

To date, the correlation between antibiotic resistance among *P. aeruginosa* clinical isolates and the production of different virulence factors is still not well understood with controversial data in the literature [19]. In addition, a few studies determined the prevalence of the different virulence factors among Egyptian clinical isolates and the role each factor plays in the development and persistence of infections [11, 20]. Identifying key virulence factors is crucial for the design of effective anti-virulence agents that can be added to the treatment of *P. aeruginosa* infections to overcome the problem of resistance development [21].

The aim of this study was to investigate the potential association between antibiotic resistance and the production of virulence factors among *P. aeruginosa* clinical isolates collected from Alexandria Main University Hospital. We also investigated the ability of phenotypic detection techniques to mirror genotypic detection of virulence factors among the tested isolates; an area infrequently studied in previous research work. Focusing on biofilm formation, as a chief virulence factor of *P. aeruginosa*, we investigated the role of alginate in the formation of biofilms by *P. aeruginosa* clinical isolates and tested the effect of ambroxol, a clinically validated mucolytic agent, on the inhibition of biofilm formation among selected isolates.

## Results

### Antibiotic susceptibility testing

**Fig. 1:**
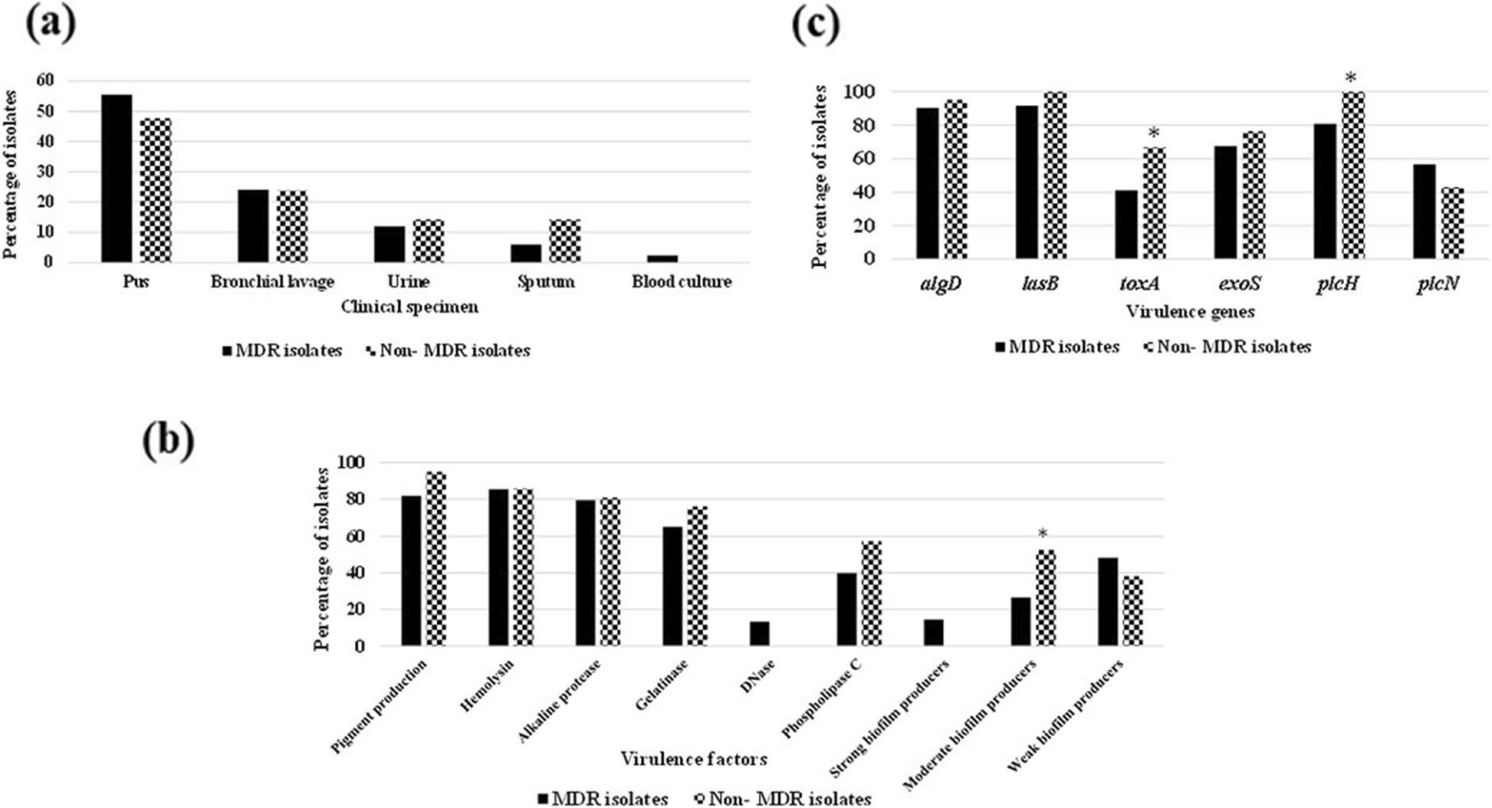
Clinical specimen distribution (**a**) and prevalence of virulence factors (**b**) and virulence genes (**c**) among MDR and non-MDR *P. aeruginosa* isolates. The *p*-values indicate significance where *p < 0.05 (significant)

The study included 104 *P. aeruginosa* clinical isolates obtained from pus (*n* = 56), bronchial lavage (*n* = 25), urine (*n* = 13), sputum (*n* = 8), and blood cultures (*n* = 2) (Additional file 1). They displayed 32 different susceptibility patterns, with only three (2.9%) isolates susceptible to all eleven tested antibiotics. Fifty-seven isolates (54.8%) were resistant to 10–11 antibiotics. Of the remaining 44 isolates resistant to < 10 antibiotics, 26 (59.1%) were multidrug-resistant (MDR), being resistant to one or more agents from the three tested classes of antibiotics [22] (Additional file 2). In total, 83 isolates (79.8%) were MDR, including both bloodstream isolates, 80% and 82.1% of bronchial lavage and pus isolates, respectively. However, no statistically significant association between the isolate clinical specimen and MDR status was observed (Fig. 1a).

**Fig. 2:**
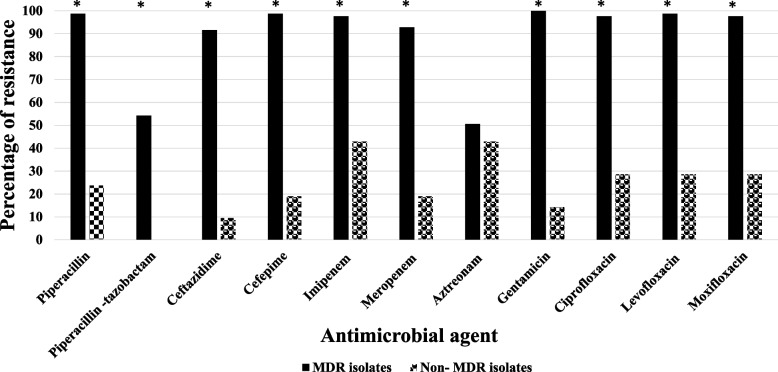
Prevalence of resistance to various antimicrobial agents among MDR and non-MDR *P. aeruginosa* clinical isolates. The *p*-values indicate significance where *p < 0.05 (significant)

The highest resistance rates among MDR isolates were to gentamicin (100%) and among non-MDR isolates were to imipenem and aztreonam (42.9% each). On the other hand, MDR isolates were most susceptible to aztreonam (49.4%) and non-MDR isolates were totally susceptible to piperacillin-tazobactam. Resistance to the tested antimicrobial agents, except aztreonam, was significantly linked to MDR isolates (Fig. 2 and Additional file 2).

The predominance of certain resistance patterns was noticeable among the tested clinical isolates. Twenty-one, out of 104, isolates (20.2%) shared the same resistance pattern where they showed resistance to the eleven tested antibiotics. Also, a common resistance pattern was observed in case of 19 isolates (18.3% of the tested isolates) which were resistant to all the tested antibiotics except for aztreonam (Additional file 2).

### Phenotypic and genotypic detection of some virulence factors and genes

**Fig. 3:**
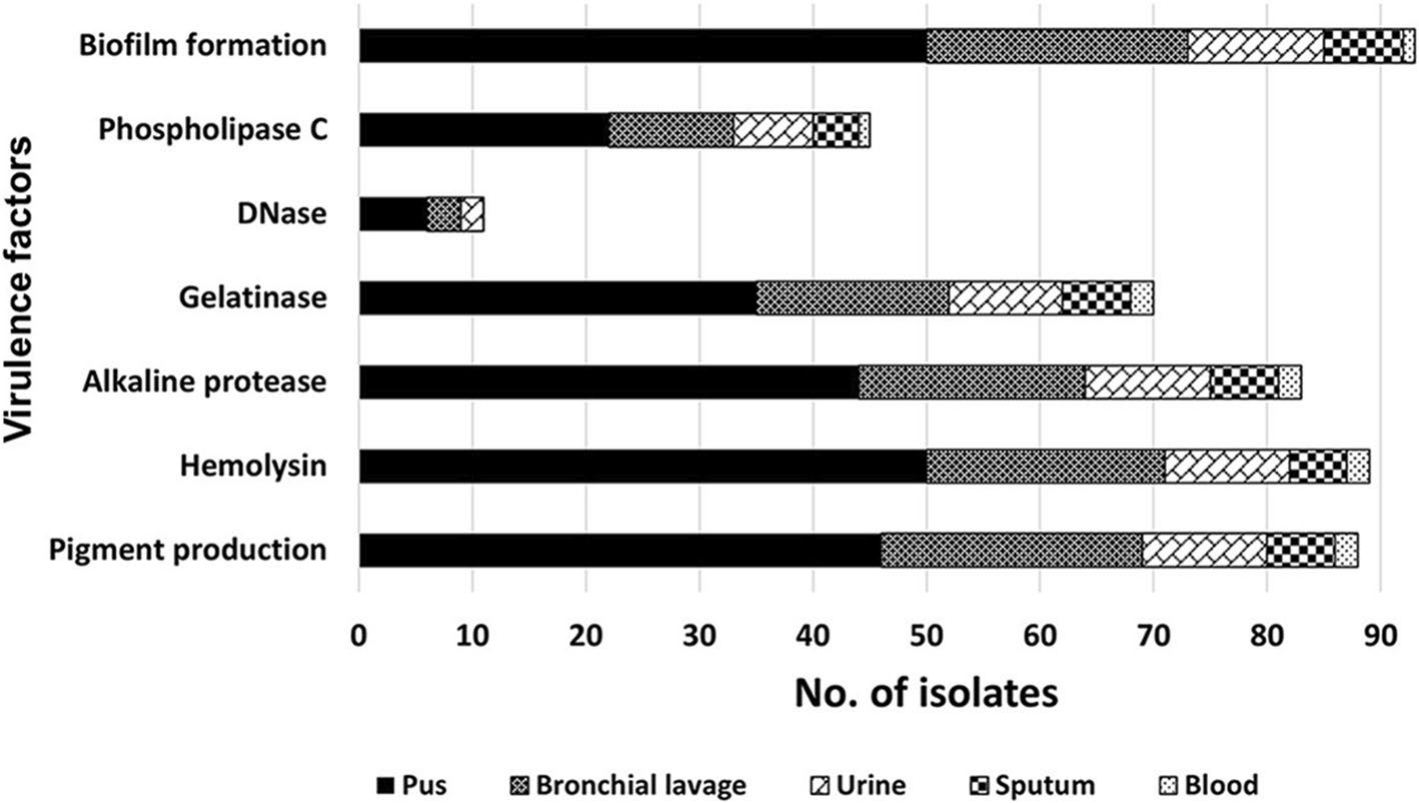
Distribution of virulence factors among the *P. aeruginosa* clinical isolates stratified by their clinical source

The most predominant virulence factor, particularly among MDR isolates, was biofilm formation (89.4%), of these 51.6%, 35.5% and 12.9% were weak, moderate, and strong biofilm producers, respectively. On the contrary, DNase was the least detected virulence factor (10.6%); it was totally absent in non-MDR isolates. With the exception of phospholipase C and gelatinase, the frequency of detection of the virulence factors among MDR and non-MDR isolates was comparable (Fig. 1b). Pigment production was observed visually where the most frequently produced pigment was pyoverdine (bright green and fluorescent) (74%), followed by pyocyanin (blue green) (9.6%), and finally pyomelanin (brown) that was detected in only one isolate. With the exception of bloodstream isolates (*n* = 2), the majority of the isolates from different clinical specimens (62.5%-92.3%) produced pigments, hemolysins, alkaline protease and gelatinase and/or formed biofilm (Fig. 3 and Additional file 1), yet the association between clinical specimen and virulence factor production was not significant. Regarding the association between the production of virulence factor and MDR phenotype, moderate biofilm formation was significantly linked to non-MDR status of the isolates (*p*-value = 0.0345**)** (Fig. 1b). Linking antibiotic susceptibility and virulence factor prevalence revealed significant association between pigment production and ceftazidime susceptibility, between phospholipase C production and cefepime susceptibility, and between DNase production and intermediate resistance to meropenem (*p*-value < 0.05) (Additional file 3).

Nineteen isolates (18.3%) possessed all six tested genes, and one isolate (1%) lacked all genes. Over half of the isolates (58.7%) harbored 4–5 genes (Additional file 1). *lasB* and *algD* showed the highest prevalence rates (93.3% and 91.3%, respectively), with no significant difference between MDR and non-MDR isolates. *toxA* was least detected among the isolates, and it was significantly associated with non-MDR isolates (*p-*value = 0.0494) and with ceftazidime susceptibility (*p*-value = 0.003). *plcH* was also significantly linked to non-MDR isolates (*p*-value = 0.037) and with susceptibility to piperacillin-tazobactam (*p*-value = 0.025). *exoS* was significantly associated with susceptibility to ceftazidime (*p*-value = 0.015) and aztreonam (*p*-value = 0.0105) (Fig. 1c and Additional file 4).

**Fig. 4:**
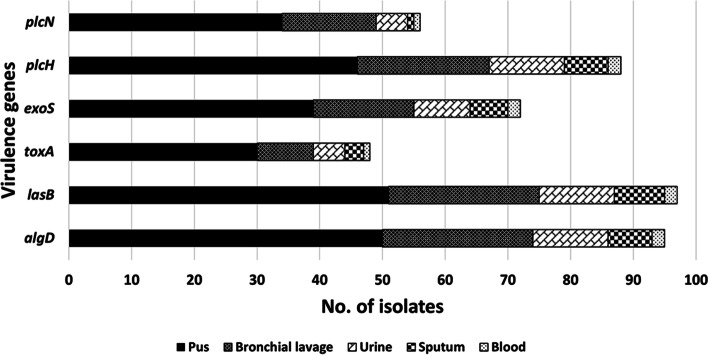
Distribution of virulence genes among the *P. aeruginosa* clinical isolates stratified by their clinical source

Both blood isolates (100%) possessed *algD*, *lasB*, *exoS* and *plcH*. The same combination of genes was also prevalent among sputum isolates at 87.5%, 100%, 75% and 87.5%, respectively. *algD*, *lasB* and *plcH* were present in over 80% of the isolates from pus, bronchial lavage and urine. However, the association between clinical specimen and virulence genes was not significant (Fig. 4).

**Fig. 5:**
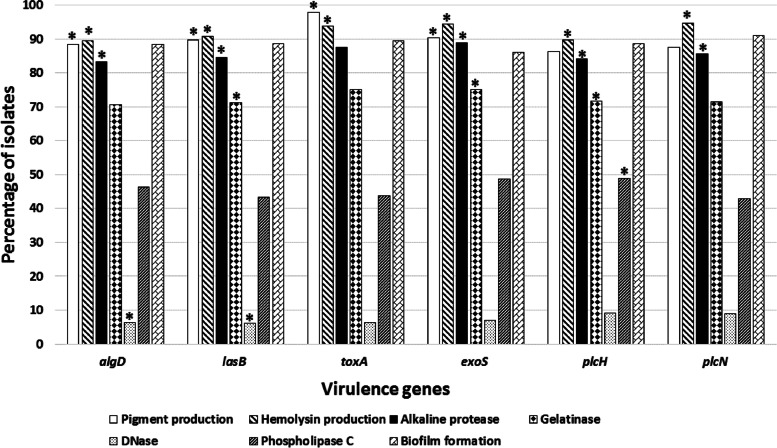
Association between the phenotypic detection of virulence factors and the presence of virulence genes among the *P. aeruginosa* clinical isolates. The *p*-values indicate significance where *p < 0.05 (significant)

Among the tested isolates, hemolysin production was significantly linked to the presence of the six tested virulence genes, while pigment production was significantly associated with the detection of *algD*, *lasB*, *toxA* and *exoS*. There was a statistically significant association between alkaline protease production and the detection of *algD*, *lasB*, *exoS*, *plcH* and *plcN*, as well as gelatinase production and the presence of *lasB*, *exoS* and *plcH*. DNase production was significantly related to the presence of *algD* and *lasB*, while phospholipase C production was significantly linked to the detection of *plcH* (Fig. 5).

The association between the detection of *algD* gene among *P. aeruginosa* clinical isolates and the biofilm formation potential of these isolates was studied among weak, moderate, and strong biofilm producers, and non-biofilm producers using the Chi-square test. A non-significant association between the presence of *algD* and the biofilm formation potential was observed among the tested isolates (*P*-value = 0.086).

### Effect of ambroxol on biofilm formation by selected *P. aeruginosa* clinical isolates

**Fig. 6:**
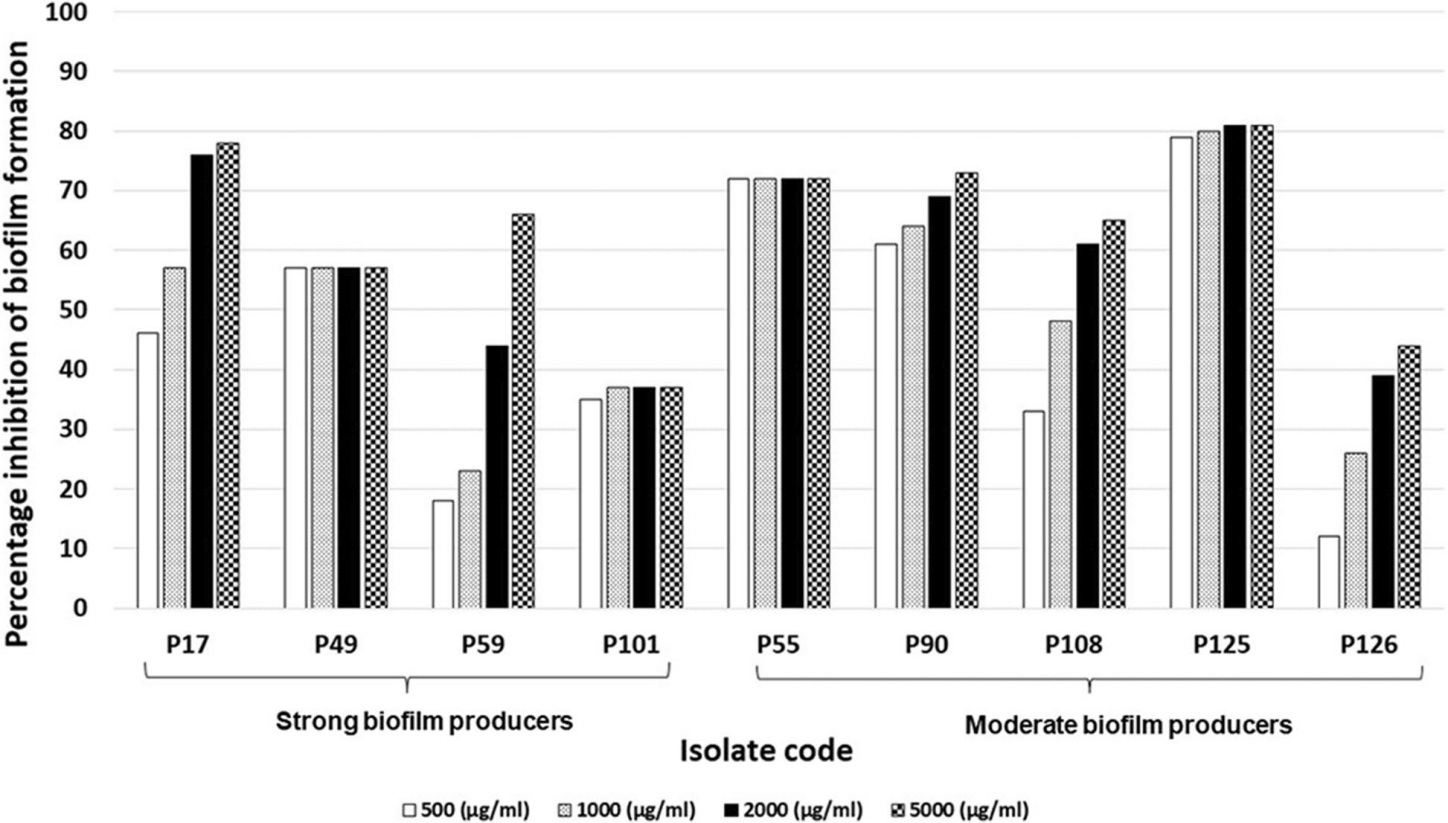
Percentage inhibition of biofilm formation among representative strong and moderate biofilm producing *P. aeruginosa* clinical isolates at different concentrations of ambroxol

The effect of ambroxol on biofilm formation among 10 strong biofilm producers and 31 moderate biofilm producers was determined. Ambroxol showed a noticeable anti-biofilm activity at all the tested concentrations. The magnitude of inhibition of biofilm formation ranged between (5%—87.5%), (20%—88%), (29%—89%) and (31% and 92%) at 500, 1000, 2000 and 5000 µg/ml of ambroxol, respectively. Figure 6 illustrates the percentage inhibition of biofilm formation among representative strong and moderate biofilm producing *P. aeruginosa* clinical isolates at different concentrations of ambroxol.

### Molecular quantification of *algD* expression by quantitative reverse transcriptase polymerase chain reaction (RT-qPCR)

**Fig. 7:**
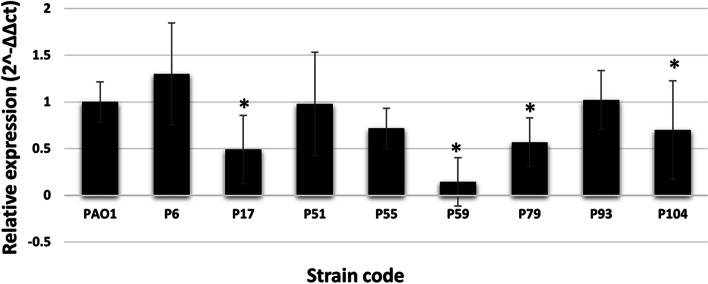
Relative expression of *algD* gene detected by RT-qPCR among selected *P. aeruginosa* clinical isolates compared to PAO1 strain. Results are expressed as the means and standard deviations of three independent determinations. Error bars represent standard deviations. The *p*-values indicate significance where *p < 0.05 (significant)

To determine the role of alginate in biofilm formation, quantification of *algD* expression in selected *P. aeruginosa* clinical isolates was determined using RT-qPCR. The isolates were selected to represent different biofilm strengths and the selection was based on their similarity in possessing different virulence factors and virulence genes. The tested isolates included strong biofilm producers (P17 & P59), moderate biofilm producers (P55 & P79), weak biofilm producers (P51 & P104) and non-biofilm producers (P6 & P93). The results showed significantly lower level of expression of *algD* gene in isolates P17, P59, P79 and P104 compared to PAO1 strain (*p*-value < 0.05), while non-significant lower level of expression was observed in case of P51 and P55 (*p*-value > 0.05). On the other hand, both non-biofilm producing isolates, P6 and P93, showed higher *algD* expression level compared to PAO1 (*p*-value > 0.05) (Fig. 7).

## Discussion

*P. aeruginosa* has become a leading cause of life-threatening Gram-negative hospital-acquired infections [23]. Moreover, emergence of MDR *P. aeruginosa* has reduced available treatment options and elevated the burden of morbidity and mortality [24]. In the current study, 79.8% of the tested *P. aeruginosa* isolates were MDR. Such widespread of MDR *P. aeruginosa* isolates is concordant with previous reports from Egypt [25, 26]. This could be attributed to the misuse of antibiotics which necessitates strict prescription policies to overcome this problem [26].

*P. aeruginosa* is equipped with numerous virulence factors which significantly contribute to its pathogenicity [6, 27]. One of these factors is pigment production that was observed among 84.6% of the tested isolates; thus, representing a higher prevalence comparable to previous studies [28–31]. Among the tested isolates, 3 pigments were detected: pyoverdine, pyocyanin and pyomelanin among 74, 9.6 and 1% of the isolates, respectively. Similarly, Alonso et al. [29] and Jácome et al. [31] reported pyoverdine and pyocyanin, respectively, to be the most detected pigments. Pyocyanin is commonly linked to tissue injury via generation of reactive oxygen species [12], pyoverdine is an important iron-chelator [29], while pyomelanin decreases the sensitivity of the microorganism to oxidative stress [32].

Hemolysin is crucial for the spread of the *P. aeruginosa* infections where it allows the invasion of the microorganism of host cells [33]. It was detected among 85.6% of our isolates. Such high frequency is in line with other studies previously conducted in Egypt [24, 28]. About 79.8% of the tested isolates possessed alkaline protease activity, involved in decomposition of complement proteins [34]. This result is in harmony with those reported in Egypt [24, 28] and Turkey [35]. Gelatinase, a protease that destroys several proteins in host cells [30], was detected among 67.3% of the tested isolates. A comparable prevalence was recorded by Georgescu et al. [36] and Pramodhini et al. [37]. DNase contributes to *P. aeruginosa* virulence through the decomposition of the extracellular DNA to be utilized as a source of nutrition [14]. DNase was the least detected virulence factor among the tested isolates (10.6%). Similarly, it was the least predominant factor detected in other studies [36, 38]. Phospholipases in *P. aeruginosa* degrade phospholipids present in pulmonary surfactants and destroy the cell membrane facilitating bacterial invasion [9]. The prevalence of phospholipase C among our isolates was comparable to that reported in Malaysia [39]. However, higher prevalence rates were detected in Romania and India [36, 37].

Besides, biofilm formation is considered a significant virulence factor related to the pathogenicity of *P. aeruginosa* during various infections, including CF, wound infections and infections related to implanted devices [40]. Among the tested isolates, 89.4% were biofilm producers. Similarly, high percentages of biofilm formation were also recorded in Egypt [24, 28]. Ambroxol has been used in cases of chronic bronchitis and bronchial asthma due to its expectorant, mucolytic and bronchosecretolytic activities. Ambroxol has shown antiadhesive properties and could prevent *P. aeruginosa* from attaching to the cultured mammalian cells [41]. Furthermore, it has been found that ambroxol can damage the structure of *P. aeruginosa* biofilm in vitro and hence reduces the lung injury related to biofilm formation in vivo. Besides, ambroxol reduces biofilm matrix in *P. aeruginosa* and interferes with quorum sensing [42, 43]. In this study, when tested for anti-biofilm activity, ambroxol showed percentage inhibition of biofilm formation ranging from 5 to 92%. In accordance with the current findings, Abbas et al. [44] reported that ambroxol possessed a noticeable effect on *P. aeruginosa* biofilms. Additionally, Lu et al. [42] demonstrated that, after treatment with ambroxol, *P. aeruginosa* biofilms noticeably decreased in extent with a decrease in the number of viable cells.

Except for pigment production association with ceftazidime susceptibility, phospholipase C production association with cefepime susceptibility, and DNase production association with intermediate resistance to meroepenem, the production of virulence factors among the tested isolates was not significantly associated with their susceptibility. Lack of association has been reported by Rodulfo et al. [30], whereas Karatuna and Yagci [45] demonstrated a significant relationship between pigment production and ceftazidime susceptibility. Also, Deptula and Gospodarek [46] reported increased phospholipase C and pigment production in multidrug-sensitive *P. aeruginosa* isolates. The prevalence of virulence factors was not significantly different between MDR and non-MDR isolates except for the association between moderate biofilm formation and non-MDR status of the isolates. In contrast, El-Mahdy and El-Kannishy [24] reported that biofilm formation was significantly associated with MDR *P. aeruginosa* isolates. As a matter of fact, the relationship between antibiotic resistance and virulence is not fully understood. However, it is believed to follow a Darwinian model where traits that confer specific advantages to the strain will be selected and become fixed whether in a positive or negative association between resistance and virulence [47].

Among the tested virulence genes, *algD* gene was detected among 91.3% of the tested isolates. In 2019, a comparative prevalence (89.4%) was detected in Egypt [48]. *algD* encodes the rate-limiting step in the production of alginate, a polysaccharide component of the biofilm matrix. Alginate is often found in the biofilms formed in the lungs of chronically colonized CF patients and is responsible for the mucoid phenotype. Also, it protects *P. aeruginosa* from host defense mechanisms through reducing phagocytosis and inhibition of activation of complement proteins [49, 50]. The obtained results indicated that the relative expression of the *algD* gene was not related to biofilm formation strength where non-biofilm producing isolates possessed higher transcription levels of *algD* than *P. aeruginosa* PAO1 and strong biofilm-producing isolates. In addition, weak biofilm producing isolates showed higher expression levels compared to strong and moderate biofilm producing isolates. It was also observed that some of *algD*-negative isolates were biofilm producers. Such observation is in agreement with Mclntyre-Smith et al. [51] who documented that alginate is not an integral matrix component in biofilm formation. Similarly, Ghadaksaz et al. [52] reported a non-significant difference in alginate production between biofilm producing and non-biofilm producing *P. aeruginosa* isolates.

Among the tested isolates, 84.6% and 53.8% harbored the phospholipases encoding genes *plcH* and *plcN,* respectively. Such results are in harmony with those obtained by Faraji et al. [53]*. lasB* gene, encoding for elastase, was the commonest (93.3%) amongst the screened genes. Comparable prevalence rates were previously reported in Morocco and Jordan [22, 54]. The gene encoding for exoenzyme S, *exoS*, was detected in 69.2% of the tested isolates; a percentage similar to that reported in an Iranian study [55]. A highly toxic virulence attribute in *P. aeruginosa*, exotoxin A, is encoded by *toxA* gene that was the least common virulence gene among our isolates (only 46.2%). In contrast, in one Egyptian study, *toxA* was the most predominant gene (95.7%) [48], while in another one, *toxA* gene was not detected at all [56]. The variability in the distribution of virulence genes among isolates may be due to their ability to adapt well to conditions present in the infection site [57]. The predominance of a certain virulence factor may be affected by various factors including the level of antibiotic resistance and mutations in virulence gene [58].

With the exception of the association of *toxA* with ceftazidime susceptibility, *exoS* with ceftazidime and aztreonam susceptibility, and *plcH* with piperacillin-tazobactam susceptibility, no significant association was noticed between the presence of virulence genes and antibiotic resistance among the tested isolates. This is in alignment with previous studies [22, 54, 59]. However, in Egypt, Sonbol et al. [56] reported a significant association between the resistance to antimicrobial agents and presence of virulence genes. In the current study, *toxA* and *plcH* presence were significantly associated with non-MDR status of the isolates. No significant association was detected between presence of the virulence genes and multidrug resistance. This finding is in accordance with those previously reported in Egypt and Iran [48, 60]. Generally, no statistical difference was detected concerning the distribution of the tested virulence factors or virulence genes among isolates obtained from different specimens. This was in accordance with what was previously recorded by Khalil et al. [28] and Wolska and Szweda [61], respectively.

In the current study, a significant association has been observed between the phenotypic detection of various tested virulence factors and the presence of certain virulence genes; a finding rarely discussed in previous research work. Among our isolates, *lasB* was significantly associated with all detected virulence factors, except for biofilm formation that was not significantly associated with any of the tested genes and phospholipase C production that was only associated with the detection of *plcH*, the gene encoding for the production of the exotoxin hemolytic phospholipase C [62].

Pigment production was associated with the detection of four out of the six genes (*algD*, *lasB*, *toxA* and *exoS*). Finlayson and Brown have reported that pigment production among* P. aeruginosa* clinical isolates was more significantly linked to the presence of virulence-associated genes, with *lasB* and *exoS* detected more frequently in pigmented isolates than in non-pigmented ones [63]. Similarly, Macin and Akyon [38] showed that pigment-producing *P. aeruginosa* clinical strains possessed significantly more *exoS* compared to non-pigmented strains.

Extracellular virulence factors such as elastases, alkaline protease, hemolysins (phospholipases and rhamnolipids) and pyocyanin pigment can play a role in the invasion step during the acute phase of *P. aeruginosa* infection. In addition, tissue damage by *P. aeruginosa* toxins can facilitate blood vessels invasion, bacterial dissemination and multiple organ failure during the acute phase of infection [5]. Many of the extracellular virulence determinants needed for tissue invasion and dissemination are regulated by quorum sensing systems which allow the pathogen to secrete such factors in a coordinated, cell-density-dependent manner and impair the host defense mechanisms [5]. This might account for the current finding that the production of each of hemolysin and alkaline protease was significantly associated with the detection of the elastase encoding gene (*lasB*), genes encoding for phospholipases C (*plcH* and *plcN*), and the toxin exoenzyme S encoding gene (*exoS*)*.*

In the current study, the presence of *algD* was not significantly associated with the biofilm formation potential among the tested isolates. Similarly, Ghadaksaz et al. [52] reported a non-significant difference in the frequency of alginate genes, including *algD*, between biofilm producing and non-biofilm producing *P. aeruginosa* isolates.

## Conclusions

In conclusion, *P. aeruginosa* clinical isolates included in our study showed high virulence as evidenced by the phenotypic and genotypic detection of virulence factors. Biofilm formation was recorded as a major virulence factor seen among the majority of the isolates. High virulence coupled with the isolates’ elevated and multi-drug resistance to commonly used antimicrobial agents would increase morbidity and mortality rates associated with *P. aeruginosa* infections. Ambroxol that displayed anti-biofilm action could be suggested (either alone or in combination with different antimicrobial agents) as alternative therapy, yet in vivo studies are required to confirm these findings. We recommend active surveillance of antimicrobial resistance and virulence determinant prevalence for better understanding of coregulatory mechanisms. This will allow an informed approach to infection control/treatment regimens.

## Methods

### Bacterial isolates

One hundred and four non-duplicate *P. aeruginosa* clinical isolates were collected from the medical microbiology lab at Alexandria Main University Hospital, Alexandria, Egypt, from September 2017 to November 2017 and from June 2018 to October 2018. They were obtained from different clinical specimens: pus, bronchial lavage, urine, sputum, and blood cultures. The collected isolates were identified using conventional techniques, such as Gram staining, cultivation on cetrimide agar plates (Himedia, India), growth at 42 °C, triple sugar iron (TSI) test (LAB M, UK), and oxidase test (Himedia, India) [64]. For long term preservation, the isolates were inoculated into 20% glycerol broth and preserved at -80 °C. For routine work, the isolates were maintained at 4 ºC on nutrient agar (Oxiod, England) plates.

### Antibiotic susceptibility testing

The susceptibility of the tested isolates to different antibiotic classes was determined by the standard disc diffusion technique, and the results were interpreted according to the Clinical and Laboratory Standards Institute (CLSI) (2018) standards [65], using Piperacillin (PI, 100 μg), Piperacillin-Tazobactam (PIT, 110 μg), Ceftazidime (CAZ, 30 μg), Cefepime (CPM, 30 μg), Imipenem (IPM, 10 μg), Meropenem (MRP, 10 μg), Aztreonam (AT, 30 μg), Gentamicin (GEN, 10 μg), Ciprofloxacin (CIP, 5 μg), Levofloxacin (LE, 5 μg), and Moxifloxacin (MO, 5 μg) discs (Himedia, India). *P. aeruginosa* ATCC 27853 was included in the experiments as a quality control strain. The MDR phenotype of the tested isolates was defined as resistance to at least three classes of antibiotics [22].

### Phenotypic detection of virulence factors

#### Pigment production

Clinical isolates were aseptically streaked on the surface of cetrimide agar plates and incubated for 24 h at 37 °C. The isolates were then visually examined for any pigment production [31].

#### Hemolysin production

The tested isolates were streaked onto the surface of 5% blood agar plates. The formation of a clear halo around the bacterial growth, after incubation for 24 h at 37 ºC, was indicative of the production of hemolysin [35].

#### Gelatin hydrolysis

Bacterial cells were inoculated via stabbing in tubes containing nutrient gelatin medium, incubated for 48 h at 37 ºC, then kept in a refrigerator or on ice for 30 min to detect the production of gelatinase. Hydrolysis of gelatin was observed as liquefaction of the medium. An uninoculated tube was included in the experiment as a negative control [66].

#### DNase production

A single colony of each isolate was streaked onto the surface of DNase agar plate (LAB M, UK). After incubation at 37 °C for 24 h, the plates were flooded with 1N HCl. The generation of a clear zone around the bacterial growth indicated DNase production [35]. *Staphylococcus aureus* ATCC 6538 was used as a positive control.

#### Alkaline protease production

The tested isolates were streaked onto the surface of skimmed milk agar plates and incubated at 37 °C for 24 h. The plates were checked for transparent zones around the bacterial growth which indicated the production of alkaline protease [28].

#### Phospholipase C production

The clinical isolates were cultivated onto the surface of egg yolk agar plates and incubated at 37 °C for 24 h. A clear zone around the bacterial growth was indicative of the phospholipase C activity [36].

#### Biofilm formation

The tested isolates were grown overnight at 37 °C in tryptic soy broth (TSB) (Himedia, India) containing 0.25% glucose, then diluted 1:100 in sterile TSB medium. Sterile flat-bottomed 96-well polystyrene microtiter plates (Citotest, China) were inoculated with 125 µl of each bacterial suspension and incubated for 24 h at 37 °C without agitation. The wells were rinsed thrice with distilled water, air dried, and stained with 125 µl of 0.1% gentian violet (Allied chemical, India) solution for 15 min. After proper washing, destaining was done using 125 μl of 30% acetic acid (El-Gomhouria Co., Egypt), then the absorbance was measured at 630 nm using the ELISA plate reader (Biotek, USA). Uninoculated medium and *P. aeruginosa* PAO1 were used as negative and positive controls, respectively. Each data point was averaged from duplicate wells. Based on the average optical density of each sample (ODs) and on the average optical density of the negative control (ODc), the samples were classified as strong biofilm producers (4xODc < ODs), moderate biofilm producers (2xODc < ODs ≤ 4xODc), weak biofilm producers (ODc < ODs ≤ 2xODc), or non-biofilm producers (ODs ≤ ODc) [67].

### Detection of genes encoding selected virulence factors

For preparation of DNA template, four colonies of each tested isolate were suspended in 200 μl of sterile water for injection. The suspension was heated at 95 °C for 30 min and then frozen at -20 °C for 30 min. After thawing, the tubes were centrifuged at 14000 rpm for 10 min, then the supernatant was aliquoted and preserved at -20 °C for future use [68, 69]. The presence of selected virulence genes, *algD, lasB, toxA, plcH, plcN* and *exoS*, was tested in the extracted DNA by the polymerase chain reaction (PCR) using previously published primers [61] (Thermo-Scientific, USA). The sequences of the used primers and the amplification conditions [53] are illustrated in Additional files 5 and 6, respectively. The amplified PCR products were resolved using 1% agarose gel electrophoresis in Tris Acetate EDTA (TAE) buffer (40 mM Tris, 20 mM acetic acid and 1 mM EDTA, pH 8.5) at 100–120 V. Following electrophoresis, the gel was visualized using a UV trans-illuminator (Entela, USA) at 254 nm. The sizes of the bands were determined relative to the loaded DNA ladder.

### Effect of ambroxol on biofilm formation

A stock solution of ambroxol (15 mg/ml) was prepared by dissolving ambroxol tablets (Ambroxol® 30 mg tablets, Glaxosmithkline) in sterile distilled water, then centrifugation at 12000 rpm for 15 min. The supernatant was filtered using cellulose acetate syringe filters (0.45 µm X 13 mm, pore size) (Filter-bio, China) and serially diluted to obtain different sub-inhibitory concentrations of ambroxol (500, 1000, 2000 and 5000 µg/ml). The effect of ambroxol on biofilm formation among selected strong and moderate biofilm producing isolates was tested using the previously described procedure. In the test, 100 µl aliquots of the overnight bacterial suspension, diluted 1:50 in double strength TSB medium, were added in duplicates to the wells of sterile 96-well polystyrene microplate containing 100 µl of sub-inhibitory ambroxol concentrations. After incubation, rinsing, staining with 0.1% gentian violet and destaining with 30% acetic acid, the OD in the presence (treated) and absence (control) of ambroxol was measured with ELISA plate reader at a wavelength of 630 nm [70], and the percentage inhibition of biofilm formation was calculated [71].

### Molecular quantification of *algD* by RT-qPCR

RT-qPCR was applied to assess the relative expression of *algD* among selected *algD*-positive *P. aeruginosa* isolates forming biofilms of different strength as well as the standard biofilm producer strain PAO1 using the Applied Biosystems 7500 Fast Real-Time PCR System (Thermo Fisher Scientific Inc., USA). The data were normalized against the housekeeping gene *rpsl*. The primers of the *rpsl* gene [16] (Willowfort, Birmingham) are shown in Additional file 5. Total RNA was extracted from the tested strains using Easy-RED™ RNA Extraction Kit (Intron, Korea) in accordance with the manufacturer’s instructions. The quantification of RNA was done using Jenway Genova nano micro-volume spectrophotometer (Kieson products, UK). To obtain the complementary DNA (cDNA), reverse transcription was accomplished using Maxime RT PreMix kit (Intron, Korea). The composition of the real-time PCR mixture was as follows: 10 μl of TOPreal™ qPCR 2X PreMix (Enznomics, South Korea), 1 μl of each primer (forward/ reverse) of both *algD* and *rpsl* genes, 1 μl of cDNA, and sterile DNase-free water made up a total reaction volume of 20 μl. The cycling conditions used for the PCR were: initial denaturation step at 95 ºC for 10 min, followed by 45 cycles of denaturation at 95 ºC for 10 s, annealing at 55 ºC for 15 s and extension at 72 ºC for 30 s. A negative control consisting of an RNA sample in RNase free water was included to check for genomic DNA contamination. Samples were tested in triplicate.

To ensure the absence of primer dimers and other artifacts, melting curves were analyzed in one cycle of 94 ºC, 53 ºC and 94 ºC, one minute each. The amplification curves, as well as the values of the cycle threshold (Ct) were established using the Stratagene MX3005P software. The level of the expression of *algD* gene was normalized to the expression level of the housekeeping gene *rpsl* and compared to the corresponding expression levels in the control *P. aeruginosa* PAO1 strain using the 2^−ΔΔ*Ct*^ method [72].

### Statistical analysis

Data were analyzed using the Chi-square and Fisher^’^s exact tests analysis using SPSS version 25 for windows (SPSS Inc., Chicago, IL, USA). One-way ANOVA was conducted for multi-variable comparison of RT-qPCR data. Differences were considered significant at *p*-value < 0.05.

## Supplementary Information

**Additional file 1.** Clinical source and virulence profile of the collected *P.* *aeruginosa* clinical isolates.

**Additional file 2.** Heat map showing the antimicrobial resistance pattern of the tested *P.* *aeruginosa* clinical isolates.

**Additional file 3**. Analysis of the association between virulence factors presence and susceptibility to antimicrobial agents.

**Additional file 4.** Analysis of the association between virulence genes and susceptibility to antimicrobial agents.

**Additional file 5.** Oligonucleotide primers sequence and amplicon size.

**Additional file 6.** PCR amplification conditions for genes encoding selected virulence factors.

## Data Availability

The datasets used and/or analyzed during the current study are available from the corresponding author on reasonable request.

## Abbreviations

MDR: Multi-drug resistant
CF: Cystic fibrosis
DNase: Deoxyribonuclease
RT-qPCR: Quantitative reverse transcriptase polymerase chain reaction
TSI: Triple sugar iron
ATCC: American Type Culture Collection
CLSI: Clinical and Laboratory Standards Institute
TSB: Tryptic soy broth
OD: Optical density
PCR: Polymerase chain reaction
EDTA: Ethylenediaminetetraacetic acid
*algD*: Alginate encoding gene
*lasB*: Elastase B encoding gene
*toxA*: Exotoxin A encoding gene
*exoS*: Exoenzyme S encoding gene
*plcH*: Phospholipase C hemolytic encoding gene
*plcN*: Phospholipase C non-hemolytic encoding gene
DNA: Deoxyribonucleic acid
TAE: Tris Acetate EDTA
UV: Ultraviolet
*rpsl*: 30S ribosomal subunit protein S12 encoding gene
cDNA: Complementary deoxyribonucleic acid
Ct: Cycle threshold

## References

[1] Ikeno T, Fukuda K, Ogawa M, Honda M, Tanabe T, Taniguchi H (2007). Small and rough colony Pseudomonas aeruginosa with elevated biofilm formation ability isolated in hospitalized patients. Microbiol Immunol.

[2] Chatterjee M, Anju C, Biswas L, Kumar VA, Mohan CG, Biswas R (2016). Antibiotic resistance in Pseudomonas aeruginosa and alternative therapeutic options. Int J Med Microbiol.

[3] Pang Z, Raudonis R, Glick BR, Lin T-J, Cheng Z (2019). Antibiotic resistance in Pseudomonas aeruginosa: mechanisms and alternative therapeutic strategies. Biotechnol Adv.

[4] Mitov I, Strateva T, Markova B (2010). Prevalence of virulence genes among Bulgarian nosocomial and cystic fibrosis isolates of Pseudomonas aeruginosa. Braz J Microbiol.

[5] Strateva T, Mitov I (2011). Contribution of an arsenal of virulence factors to pathogenesis of Pseudomonas aeruginosa infections. Ann Microbiol.

[6] Lau GW, Hassett DJ, Britigan BE (2005). Modulation of lung epithelial functions by Pseudomonas aeruginosa. Trends Microbiol.

[7] Hauser AR (2009). The type III secretion system of Pseudomonas aeruginosa: infection by injection. Nat Rev Microbiol.

[8] Shaver CM, Hauser AR (2004). Relative contributions of Pseudomonas aeruginosa ExoU, ExoS, and ExoT to virulence in the lung. Infect Immun.

[9] Gellatly SL, Hancock RE (2013). Pseudomonas aeruginosa: new insights into pathogenesis and host defenses. Pathog Dis.

[10] López DJ, Collado MI, Ibarguren M, Vasil AI, Vasil ML, Goñi FM (2011). Multiple phospholipid substrates of phospholipase C/sphingomyelinase HR2 from Pseudomonas aeruginosa. Chem Phys Lipids.

[11] Hassuna NA, Mandour SA, Mohamed ES (2020). Virulence constitution of multi-drug-resistant Pseudomonas aeruginosa in Upper Egypt. Infect Drug Resist.

[12] Muller M (2002). Pyocyanin induces oxidative stress in human endothelial cells and modulates the glutathione redox cycle. Free Radic Biol Med.

[13] Allen L, Dockrell DH, Pattery T, Lee DG, Cornelis P, Hellewell PG (2005). Pyocyanin production by Pseudomonas aeruginosa induces neutrophil apoptosis and impairs neutrophil-mediated host defenses in vivo. J Immunol.

[14] Mulcahy H, Charron-Mazenod L, Lewenza S (2010). Pseudomonas aeruginosa produces an extracellular deoxyribonuclease that is required for utilization of DNA as a nutrient source. Environ Microbiol.

[15] Alnour TM, Ahmed-Abakur EH (2017). Multidrug resistant Pseudomonas (P) aeruginosa: medical impact, pathogenicity, resistance mechanisms and epidemiology. JSM Microbiol.

[16] Jazayeri J, Nguyen KD, Schneiders F, Tan C (2016). Comparison of virulence factors in Pseudomonas aeruginosa strains isolated from cystic fibrosis patients. J Med Microb Diagn.

[17] Leid JG (2009). Bacterial biofilms resist key host defenses. Microbe.

[18] Chen L, Wen Ym (2011). The role of bacterial biofilm in persistent infections and control strategies. Int J Oral Sci.

[19] Persyn E, Sassi M, Aubry M, Broly M, Delanou S, Asehnoune K (2019). Rapid genetic and phenotypic changes in Pseudomonas aeruginosa clinical strains during ventilator-associated pneumonia. Sci Rep.

[20] Abd El-Baky RM, Mandour SA, Ahmed EF, Hashem ZS, Sandle T, Mohamed DS. Virulence profiles of some *Pseudomonas aeruginosa* clinical isolates and their association with the suppression of Candida growth in polymicrobial infections. PLoS ONE. 2020;15(12):e0243418. 10.1371/journal.pone.0243418 PMC7723275 33290412

[21] Khayat MT, Ibrahim TS, Khayyat AN, Alharbi M, Shaldam MA, Mohammad KA (2022). Sodium citrate alleviates virulence in Pseudomonas aeruginosa. Microorganisms.

[22] Elmouaden C, Laglaoui A, Ennanei L, Bakkali M, Abid M (2019). Virulence genes and antibiotic resistance of Pseudomonas aeruginosa isolated from patients in the Northwestern of Morocco. J Infect Dev Ctries.

[23] Wang H, Tu F, Gui Z, Lu X, Chu W (2013). Antibiotic resistance profiles and quorum sensing-dependent virulence factors in clinical isolates of Pseudomonas aeruginosa. Indian J Microbiol.

[24] El-Mahdy R, El-Kannishy G (2019). Virulence factors of carbapenem-resistant Pseudomonas aeruginosa in hospital-acquired infections in Mansoura. Egypt Infect Drug Resist.

[25] Diab M, Fam N, El-Said M, El-Dabaa E, El-Defrawy I, Saber M (2013). Occurrence of VIM-2 Metallo-β-Lactamases in imipenem resistant and susceptible Pseudomonas aeruginosa clinical isolates from Egypt. Afr J Microbiol Res.

[26] Abbas HA, El-Ganiny AM, Kamel HA (2018). Phenotypic and genotypic detection of antibiotic resistance of Pseudomonas aeruginosa isolated from urinary tract infections. Afr Health Sci.

[27] Lyczak JB, Cannon CL, Pier GB (2000). Establishment of Pseudomonas aeruginosa infection: lessons from a versatile opportunist. Microbes Infect.

[28] Khalil MA, Sonbol FI, Mohamed AB, Ali SS (2015). Comparative study of virulence factors among ESβL-producing and nonproducing Pseudomonas aeruginosa clinical isolates. Turk J Med Sci.

[29] Alonso B, Fernández-Barat L, Di Domenico EG, Marín M, Cercenado E, Merino I (2020). Characterization of the virulence of Pseudomonas aeruginosa strains causing ventilator-associated pneumonia. BMC Infect Dis.

[30] Rodulfo H, Arcia A, Hernández A, Michelli E, Martinez DDV, Guzman M (2019). Virulence factors and integrons are associated with MDR and XDR phenotypes in nosocomial strains of Pseudomonas aeruginosa in a Venezuelan university hospital. Rev Inst Med Trop Sao Paulo.

[31] Jácome PR, Alves LR, Cabral AB, Lopes AC, Maciel MA. Phenotypic and molecular characterization of antimicrobial resistance and virulence factors in *Pseudomonas aeruginosa* clinical isolates from Recife, State of Pernambuco, Brazil. Rev Soc Bras Med Trop. 2012;45(6):707-12. 10.1590/s0037-86822012000600010 23295873

[32] Ketelboeter LM, Potharla VY, Bardy SL (2014). NTBC treatment of the pyomelanogenic Pseudomonas aeruginosa clinical isolate PA1111 inhibits pigment production and increases sensitivity to oxidative stress. Curr Microbiol.

[33] Goebel W, Chakraborty T, Kreft J (1988). Bacterial hemolysins as virulence factors. Antonie Van Leeuwenhoek.

[34] Laarman AJ, Bardoel BW, Ruyken M, Fernie J, Milder FJ, van Strijp JA (2012). Pseudomonas aeruginosa alkaline protease blocks complement activation via the classical and lectin pathways. J Immunol.

[35] Macin S, Akarca M, Sener B, Akyon Y (2017). Comparison of virulence factors and antibiotic resistance of Pseudomonas aeruginosa strains isolated from patients with and without cystic fibrosis. Rev Romana Med Lab.

[36] Georgescu M, Gheorghe I, Curutiu C, Lazar V, Bleotu C, Chifiriuc M-C (2016). Virulence and resistance features of Pseudomonas aeruginosa strains isolated from chronic leg ulcers. BMC Infect Dis.

[37] Pramodhini S, Umadevi S, Seetha K (2016). Detection of virulence determinants and its association with drug resistance in clinical isolates of Pseudomonas aeruginosa. Int J Res Med Sci.

[38] Macin S, Akyon Y (2017). Phenotypic and genotypic virulence factors in Pseudomonas aeruginosa strains according to pigment presence. Acta Med Mediterr.

[39] Fazlul M, Najnin A, Farzana Y, Rashid M, Deepthi S, Srikumar C (2018). Detection of virulence factors and β lactamase encoding genes among the clinical isolates of Pseudomonas aeruginosa. Int J Pharm Res.

[40] Rybtke M, Hultqvist LD, Givskov M, Tolker-Nielsen T (2015). Pseudomonas aeruginosa biofilm infections: community structure, antimicrobial tolerance and immune response. J Mol Biol.

[41] Hafez MM, Aboulwafa MM, Yassien MA, Hassouna NA (2009). Activity of some mucolytics against bacterial adherence to mammalian cells. Appl Biochem Biotechnol.

[42] Lu Q, Yu J, Yang X, Wang J, Wang L, Lin Y (2010). Ambroxol interferes with Pseudomonas aeruginosa quorum sensing. Int J Antimicrob Agents.

[43] Li F, Yu J, Yang H, Wan Z, Bai D (2008). Effects of ambroxol on alginate of mature Pseudomonas aeruginosa biofilms. Curr Microbiol.

[44] Abbas HA, Serry FM, EL-Masry EM (2012). Combating Pseudomonas aeruginosa biofilms by potential biofilm inhibitors. Asian J Res Pharm Sci.

[45] Karatuna O, Yagci A (2010). Analysis of quorum sensing-dependent virulence factor production and its relationship with antimicrobial susceptibility in Pseudomonas aeruginosa respiratory isolates. Clin Microbiol Infect.

[46] Deptuła A, Gospodarek E (2010). Reduced expression of virulence factors in multidrug-resistant Pseudomonas aeruginosa strains. Arch Microbiol.

[47] Beceiro A, Tomás M, Bou G (2013). Antimicrobial resistance and virulence: a successful or deleterious association in the bacterial world?. Clin Microbiol Rev.

[48] Elmaraghy N, Abbadi S, Elhadidi G, Hashem A, Yousef A (2019). Virulence genes in Pseudomonas aeruginosa strains isolated at Suez Canal University Hospitals with respect to the site of infection and antimicrobial resistance. Int J Clin Microbiol Biochem Technol.

[49] Balasubramanian D, Kong K, Jayawardena SR, Leal SM, Sautter RT, Mathee K (2011). Co-regulation of β-lactam resistance, alginate production and quorum sensing in Pseudomonas aeruginosa. J Med Microbiol.

[50] Bystrova OV, Knirel YA, Lindner B, Kocharova NA, Kondakova AN, Zahringer U (2006). Structures of the core oligosaccharide and O-units in the R- and SR-type lipopolysaccharides of reference strains of Pseudomonas aeruginosa O-serogroups. FEMS Immunol Med Microbiol.

[51] McIntyre-Smith A, Schneiderman J, Zhou K (2010). Alginate does not appear to be essential for biofilm production by PAO1 Pseudomonas aeruginosa. JEMI.

[52] Ghadaksaz A, Fooladi AAI, Hosseini HM, Amin M (2015). The prevalence of some Pseudomonas virulence genes related to biofilm formation and alginate production among clinical isolates. J Appl Biomed.

[53] Faraji F, Mahzounieh M, Ebrahimi A, Fallah F, Teymournejad O, Lajevardi B (2016). Molecular detection of virulence genes in Pseudomonas aeruginosa isolated from children with cystic fibrosis and burn wounds in Iran. Microb Pathog.

[54] Al Dawodeyah HY, Obeidat N, Abu-Qatouseh LF, Shehabi AA (2018). Antimicrobial resistance and putative virulence genes of Pseudomonas aeruginosa isolates from patients with respiratory tract infection. Germs.

[55] Fazeli N, Momtaz H (2014). Virulence gene profiles of multidrug-resistant Pseudomonas aeruginosa isolated from Iranian hospital infections. Iran Red Crescent Med J.

[56] Sonbol FI, Khalil MA, Mohamed AB, Ali SS (2015). Correlation between antibiotic resistance and virulence of Pseudomonas aeruginosa clinical isolates. Turk J Med Sci.

[57] Lanotte P, Watt S, Mereghetti L, Dartiguelongue N, Rastegar-Lari A, Goudeau A (2004). Genetic features of Pseudomonas aeruginosa isolates from cystic fibrosis patients compared with those of isolates from other origins. J Med Microbiol.

[58] Dehbashi S, Tahmasebi H, Arabestani MR (2018). Association between Beta-lactam antibiotic resistance and virulence factors in AmpC producing clinical strains of P. aeruginosa. Osong Public Health Res Perspect.

[59] Amirmozafari N, Fallah MJ, Habibi A (2016). Association of the exotoxin A and exoenzyme S with antimicrobial resistance in Pseudomonas aeruginosa strains. Arch Iran Med.

[60] Najafi K, Kafil HS, Shokrian S, Azimi S, Asgharzadeh M, Yousefi M (2015). Virulence genes and antibiotic resistance profile of Pseudomonas aeruginosa isolates in Northwest of Iran. J Pure Appl Microbiol.

[61] Wolska K, Szweda P (2009). Genetic features of clinical Pseudomonas aeruginosa strains. Pol J Microbiol.

[62] Wolfmeier H, Wardell SJT, Liu LT, Falsafi R, Draeger A, Babiychuk EB (2022). Targeting the Pseudomonas aeruginosa virulence factor phospholipase C with engineered liposomes. Front Microbiol.

[63] Finlayson EA, Brown PD (2011). Comparison of antibiotic resistance and virulence factors in pigmented and non-pigmented Pseudomonas aeruginosa. West Indian Med J.

[64] Krieg NR, Holt JG. Bergey’s Manual for Systematic Bacteriology, Vol. 2: Baltimore, MD, USA: Williams and Wilkins; 2001.

[65] Clinical and Laboratory Standards Institute. Performance standards for antimicrobial susceptibility testing. CLSI. Wayne, PA; 2018.

[66] Stehling EG, Silveira WD, Leite DS (2008). Study of biological characteristics of Pseudomonas aeruginosa strains isolated from patients with cystic fibrosis and from patients with extra-pulmonary infections. Braz J Infect Dis.

[67] O'Toole GA (2011). Microtiter dish biofilm formation assay. J Vis Exp.

[68] Gupta R, Malik A, Rizvi M, Ahmed M. Presence of metallo-beta-lactamases (MBL), extended-spectrum beta-lactamase (ESBL) & AmpC positive non-fermenting Gram-negative bacilli among intensive care unit patients with special reference to molecular detection of *bla*_CTX__-M_ & *bla*_AmpC_ genes. Indian J Med Res. 2016;144(2):271–5. 10.4103/0971-5916.195043 PMC5206880 27934808

[69] Sepehriseresht S, Boroumand MA, Pourgholi L, Sotoudeh AM, Habibi E, Sattarzadeh TM (2012). Detection of vim-and ipm-type metallo-beta-lactamases in Pseudomonas aeruginosa clinical isolates. Arch Iran Med.

[70] Jadhav S, Shah R, Bhave M, Palombo EA (2013). Inhibitory activity of yarrow essential oil on Listeria planktonic cells and biofilms. Food Control.

[71] Das MC, Sandhu P, Gupta P, Rudrapaul P, De UC, Tribedi P (2016). Attenuation of Pseudomonas aeruginosa biofilm formation by vitexin: a combinatorial study with azithromycin and gentamicin. Sci Rep.

[72] Livak KJ, Schmittgen TD (2001). Analysis of relative gene expression data using real-time quantitative PCR and the 2^− ΔΔCT^ method. Methods.

