# Supplementary material for: Prevalence of different virulence factors and their association with antimicrobial resistance among Pseudomonas aeruginosa clinical isolates from Egypt
Source: BMC Microbiol. 2023 Jun 3;23:161. doi: 10.1186/s12866-023-02897-8 (PMC10239191; doi:10.1186/s12866-023-02897-8)
Supplement: Supplementary file 3 — Additional file 3. Analysis of the association between virulence factors presence and susceptibility to antimicrobial agents. [file 12866_2023_2897_MOESM3_ESM.docx]

**Additional file 3:** Analysis of the association between virulence factors presence and susceptibility to antimicrobial agents.

| Antimicrobial agent | **S *^a^*, I  *^b^* or R *^c^***  **(no.)** | | | No. of isolates producing the tested virulence factors (%) | | | | | | |
| --- | --- | --- | --- | --- | --- | --- | --- | --- | --- | --- |
|  |  |  |  | **Pigment production** | **Hemolysin** | **Alkaline protease** | **Gelatinase** | **DNase** | **Phospholipase C** | **Biofilm formation** |
| Piperacillin | **S** | 17 | | 17 (100) | 16 (94.1) | 16 (94.1) | 15 (88.2) | 0 (0) | 11 (64.7) | 15 (88.2) |
|  | **I** | 0 | | 0 (0) | 0 (0) | 0 (0) | 0 (0) | 0 (0) | 0 (0) | 0 (0) |
|  | **R** | 87 | | 71 (81.6) | 73 (83.9) | 67 (77) | 55 (63.2) | 11 (12.6) | 34 (39.1) | 78 (89.7) |
| *p*-value * | | | | **0.0676** | **0.456** | **0.184** | **0.051** | **0.204** | **0.064** | **1** |
| Piperacillin-tazobactam | **S** | 29 | | 26 (89.7) | 25 (86.2) | 23 (79.3) | 21 (72.4) | 1 (3.4) | 15 (51.7) | 26 (89.7) |
|  | **I** | 30 | | 27 (90) | 29 (96.7) | 27 (90) | 21 (70) | 4 (13.3) | 15 (50) | 27 (90) |
|  | **R** | 45 | | 35 (77.8) | 35 (77.8) | 33 (73.3) | 28 (62.2) | 6 (13.3) | 15 (33.3) | 40 (88.9) |
| *p*-value | | | | **0.240** | **0.074** | **0.211** | **0.615** | **0.339** | **0.201** | **0.987** |
| Ceftazidime | **S** | 26 | | 26 (100) | 24 (92.3) | 24 (92.3) | 21 (80.8) | 2 (7.7) | 14 (53.8) | 24 (92.3) |
|  | **I** | 0 | | 0 (0) | 0 (0) | 0 (0) | 0 (0) | 0 (0) | 0 (0) | 0 (0) |
|  | **R** | 78 | | 62 (79.5) | 65 (83.3) | 59 (75.6) | 49 (62.8) | 9 (11.5) | 31 (39.7) | 69 (88.5) |
| *p*-value | | | | **0.0102** | **0.342** | **0.0908** | **0.146** | **0.727** | **0.255** | **0.726** |
| Cefepime | **S** | 16 | | 15 (93.8) | 14 (87.5) | 13 (81.3) | 13 (81.3) | 0 (0) | 12 (75) | 15 (93.8) |
|  | **I** | 2 | | 2 (100) | 2 (100) | 2 (100) | 2 (100) | 0 (0) | 1 (50) | 2 (100) |
|  | **R** | 86 | | 71 (82.6) | 73 (84.9) | 68 (79.1) | 55 (64) | 11 (12.8) | 32 (37.2) | 76 (88.4) |
| *p-value* | | | | **0.434** | **0.811** | **0.757** | **0. 244** | **0.276** | **0.019** | **0.721** |
| Imipenem | **S** | | 13 | 13 (100) | 12 (92.3) | 12 (92.3) | 11 (84.6) | 0 (0) | 6 (46.2) | 12 (92.3) |
|  | **I** | | 1 | 1 (100) | 1 (100) | 1 (100) | 1 (100) | 0 (0) | 0 (0) | 1 (100) |
|  | **R** | | 90 | 74 (82.2) | 76 (84.4) | 70 (77.8) | 58 (64.4) | 11 (12.2) | 39 (43.3) | 80 (88.9) |
| *p*-value | | | | **0.230** | **0.691** | **0.418** | **0.274** | **0.384** | **0.668** | **0.878** |
| Meropenem | **S** | | 19 | 18 (94.7) | 17 (89.5) | 17 (89.5) | 16 (84.2) | 1 (5.3) | 9 (47.4) | 17 (89.5) |
|  | **I** | | 4 | 2 (50) | 2 (50) | 2 (50) | 2 (50) | 2 (50) | 1(25) | 4 (100) |
|  | **R** | | 81 | 68 (84) | 70 (86.4) | 64 (79) | 52 (64.2) | 8 (9.9) | 35 (43.2) | 72 (88.9) |
| *p*-value | | | | **0.074** | **0.112** | **0.188** | **0.186** | **0.028** | **0.714** | **0.780** |
| Aztreonam | **S** | | 53 | 45 (84.9) | 44 (83) | 40 (75.5) | 34 (64.2) | 4 (7.5) | 23 (43.3) | 47 (88.7) |
|  | **I** | | 0 | 0 (0) | 0 (0) | 0 (0) | 0 (0) | 0 (0) | 0 (0) | 0 (0) |
|  | **R** | | 51 | 43 (84.3) | 45 (88.2) | 43 (84.3) | 36 (70.6) | 7 (13.7) | 22 (43.1) | 46 (90.2) |
| *p*-value | | | | **1** | **0.579** | **0.331** | **0.535** | **0.354** | **1** | **1** |
| Gentamicin | **S** | | 18 | 17 (94.4) | 15 (83.3) | 15 (83.3) | 14 (77.8) | 0 (0) | 10 (55.6) | 17 (94.4) |
|  | **I** | | 0 | 0 (0) | 0 (0) | 0 (0) | 0 (0) | 0 (0) | 0 (0) | 0 (0) |
|  | **R** | | 86 | 71 (82.6) | 74 (86) | 68 (79.1) | 56 (65.1) | 11 (12.8) | 35 (40.7) | 76 (88.3) |
| *p*-value | | | | **0.294** | **0.721** | **1** | **0.410** | **0.205** | **0.23** | **0.685** |
| Ciprofloxacin | **S** | | 17 | 16 (94.1) | 15 (88.2) | 15 (88.2) | 14 (82.4) | 1 (5.9) | 9 (52.9) | 15 (88.2) |
|  | **I** | | 0 | 0 (0) | 0 (0) | 0 (0) | 0 (0) | 0 (0) | 0 (0) | 0 (0) |
|  | **R** | | 87 | 72 (82.8) | 74 (85.1) | 68 (78.2) | 56 (64.4) | 10 (11.5) | 36 (41.4) | 78 (89.7) |
| *p*-value | | | | **0.461** | **1** | **0.513** | **0.171** | **0.687** | **0.429** | **1** |
| Levofloxacin | **S** | | 16 | 15 (93.8) | 14 (87.5) | 14 (87.5) | 13 (81.3) | 0 (0) | 8 (50) | 15 (93.8) |
|  | **I** | | 0 | 0 (0) | 0 (0) | 0 (0) | 0 (0) | 0 (0) | 0 (0) | 0 (0) |
|  | **R** | | 88 | 73 (83) | 75 (85.2) | 69 (78.4) | 57 (64.8) | 11(12.5) | 37 (42) | 78 (88.6) |
| *p*-value | | | | **0.456** | **1** | **0.516** | **0.254** | **0.207** | **0.592** | **1** |
| Moxifloxacin | **S** | | 17 | 16 (94.1) | 15 (88.2) | 15 (88.2) | 14 (82.4) | 1 (5.9) | 9 (52.9) | 15 (88.2) |
|  | **I** | | 0 | 0 (0) | 0 (0) | 0 (0) | 0 (0) | 0 (0) | 0 (0) | 0 (0) |
|  | **R** | | 87 | 72 (82.8) | 74 (85.1) | 68 (78.2) | 56 (64.4) | 10 (11.5) | 36 (41.4) | 78 (89.7) |
| *p*-value | | | | **0.461** | **1** | **0.513** | **0.172** | **0.687** | **0.429** | **1** |

***^a^* S:** Sensitive. *^b^* **I:** Intermediate. ***^c^* R:** Resistant.

* ***p*-value:** < 0.05 was considered significant. Significant associations are highlighted in grey.
