# Supplementary material for: Prevalence of different virulence factors and their association with antimicrobial resistance among Pseudomonas aeruginosa clinical isolates from Egypt
Source: BMC Microbiol. 2023 Jun 3;23:161. doi: 10.1186/s12866-023-02897-8 (PMC10239191; doi:10.1186/s12866-023-02897-8)
Supplement: Supplementary file 4 — Additional file 4. Analysis of the association between virulence genes and susceptibility to antimicrobial agents. [file 12866_2023_2897_MOESM4_ESM.docx]

**Additional file 4:** Analysis of the association between virulence genes and susceptibility to antimicrobial agents.

| Antimicrobial agent | S *^a^*, I  *^b^* or R *^c^*  (no.) | | | | No. of isolates harboring virulence factors encoding genes (%) | | | | | |
| --- | --- | --- | --- | --- | --- | --- | --- | --- | --- | --- |
|  |  |  |  |  | ***algD*** | ***lasB*** | ***toxA*** | ***exoS*** | ***plcH*** | ***plcN*** |
| Piperacillin | **S** | | 17 | | 16 (94.1) | 17 (100) | 11 (64.7) | 14 (82.4) | 17 (100) | 7 (41.2) |
|  | **I** | | 0 | | 0 (0) | 0 (0) | 0 (0) | 0 (0) | 0 (0) | 0 (0) |
|  | **R** | | 87 | | 79 (90.8) | 80 (92) | 37 (42.5) | 58 (66.7) | 71 (81.6) | 49 (56.3) |
| *p*-value * | | | | | **1** | **0.596** | **0.115** | **0.258** | **0.068** | **0.295** |
| Piperacillin-tazobactam | **S** | | 29 | | 27 (93.1) | 28 (96.6) | 17 (58.6) | 22 (75.9) | 29 (100) | 11 (37.9) |
|  | **I** | | 30 | | 29 (96.7 ) | 29 (96.7) | 12 (40) | 24 (80) | 24 (80) | 18 (60) |
|  | **R** | | 45 | | 39 (86.7) | 40 (88.9) | 19 (42.2) | 26 (57.8) | 35 (77.8) | 27 (60) |
| *p*-value | | | | | **0.296** | **0.297** | **0.279** | **0.082** | **0.025** | **0.129** |
| Ceftazidime | **S** | | 26 | | 25 (96.2) | 26 (100) | 19 (73.1) | 23 (88.5) | 24 (92.3) | 13 (50) |
|  | **I** | | 0 | | 0 (0) | 0 (0) | 0 (0) | 0 (0) | 0 (0) | 0 (0) |
|  | **R** | | 78 | | 70 (89.7) | 71 (91) | 29 (37.2) | 49 (62.8) | 64 (82.1) | 43 (55.1) |
| *p*-value | | | | | **0.444** | **0.188** | **0.003** | **0.015** | **0.347** | **0.658** |
| Cefepime | **S** | | 16 | | 15 (93.7) | 16 (100) | 9 (56.3) | 11 (68.8) | 16 (100) | 6 (37.5) |
|  | **I** | | 2 | | 2 (100) | 2 (100) | 1 (50) | 1 (50) | 2 (100) | 1 (50) |
|  | **R** | | 86 | | 78 (90.7) | 79 (91.9) | 38 (44.2) | 60 (69.8) | 70 (81.4) | 49 (57) |
| *p*-value | | | | | **0.839** | **0.456** | **0.670** | **0.835** | **0.138** | **0.355** |
| Imipenem | **S** | | 13 | | 12 (92.3) | 13 (100) | 8 (61.5) | 10 (76.9) | 13 (100) | 5 (38.5) |
|  | **I** | | 1 | | 1 (100) | 1 (100) | 1 (100) | 1 (100) | 1 (100) | 0 (0) |
|  | **R** | | 90 | | 82 (91.1) | 83 (92.2) | 39 (43.3) | 61 (67.8) | 74 (82.2) | 51 (56.7) |
| *p*-value | | | | | **0.944** | **0.558** | **0.260** | **0.639** | **0.230** | **0.260** |
| Meropenem | **S** | | 19 | | 17 (89.5) | 19 (100) | 11 (57.9) | 14 (73.7) | 19 (100) | 8 (42.1) |
|  | **I** | | 4 | | 3 (75) | 3 (75) | 2 (50) | 2 (50) | 4 (100) | 3 (75) |
|  | **R** | | 81 | | 75 (92.6) | 75 (92.6) | 35 (43.2) | 56 (69.1) | 65 (80.2) | 45 (55.6) |
| *p*-value | | | | | **0.450** | **0.169** | **0.507** | **0.647** | **0.068** | **0.393** |
| Aztreonam | **S** | | 53 | | 48 (90.6) | 50 (94.3) | 27 (50.9) | 43 (81.1) | 46 (86.8) | 30 (56.6) |
|  | **I** | | 0 | | 0 (0) | 0 (0) | 0 (0) | 0 (0) | 0 (0) | 0 (0) |
|  | **R** | | 51 | | 47 (92.2) | 47 (92.2) | 21 (41.2) | 29 (56.9) | 42 (82.4) | 26 (51) |
| *p*-value | | | | | **1** | **0.713** | **0.333** | **0.0105** | **0.594** | **0.694** |
| Gentamicin | **S** | | 18 | | 17 (94.4) | 18 (100) | 12 (66.7) | 13 (72.2) | 18 (100) | 7 (38.9) |
|  | **I** | | 0 | | 0 (0) | 0 (0) | 0 (0) | 0 (0) | 0 (0) | 0 (0) |
|  | **R** | | 86 | | 78 (90.7) | 79 (91.9) | 36 (41.9) | 59 (68.6) | 70 (81.4) | 49 (57) |
| *p*-value | | | | | **1** | **0.602** | **0.070** | **1** | **0.068** | **0.198** |
| Ciprofloxacin | **S** | | 17 | | 15 (88.2) | 17 (100) | 10 (58.8) | 13 (76.5) | 17 (100) | 8 (47.1) |
|  | **I** | | 0 | | 0 (0) | 0 (0) | 0 (0) | 0 (0) | 0 (0) | 0 (0) |
|  | **R** | | 87 | | 80 (92) | 80 (92) | 38 (43.7) | 59 (67.8) | 71 (81.6) | 48 (55.2) |
| *p*-value | | | | | **0.638** | **0.595** | **0.295** | **0.576** | **0.0676** | **0.601** |
| Levofloxacin | **S** | | 16 | | 15 (93.8) | 16 (100) | 9 (56.3) | 11 (68.8) | 15 (93.8) | 6 (37.5) |
|  | **I** | | 0 | | 0 (0) | 0 (0) | 0 (0) | 0 (0) | 0 (0) | 0 (0) |
|  | **R** | | 88 | | 80 (90.9) | 81 (92) | 39 (44.3) | 61 (69.3) | 73 (83) | 50 (56.8) |
| *p*-value | | | | | **1** | **0.592** | **0.423** | **1** | **0.456** | **0.181** |
| Moxifloxacin | | **S** | | 17 | 15 (88.2) | 17 (100) | 10 (58.8) | 13 (76.5) | 17 (100) | 8 (47.1) |
|  |  | **I** | | 0 | 0 (0) | 0 (0) | 0 (0) | 0 (0) | 0 (0) | 0 (0) |
|  |  | **R** | | 87 | 80 (92) | 80 (92) | 38 (43.7) | 59 (67.8) | 71 (81.6) | 48 (55.2) |
| *p*-value | | | | | **0.638** | **0.595** | **0.295** | **0.576** | **0.0676** | **0.601** |

***^a^* S:** Sensitive. *^b^* **I:** Intermediate. ***^c^* R:** Resistant.

* ***p*-value:** < 0.05 was considered significant. Significant associations are highlighted in grey.
