# Supplementary material for: Prevalence of different virulence factors and their association with antimicrobial resistance among Pseudomonas aeruginosa clinical isolates from Egypt
Source: BMC Microbiol. 2023 Jun 3;23:161. doi: 10.1186/s12866-023-02897-8 (PMC10239191; doi:10.1186/s12866-023-02897-8)
Supplement: Supplementary file 5 — Additional file 5. Oligonucleotide primers sequence and amplicon size. [file 12866_2023_2897_MOESM5_ESM.docx]

**Additional file 5:** Oligonucleotide primers sequence and amplicon size.

| Target gene | Primer name |  | Nucleotide sequence (5′→3′) | Size of the  amplicons (bps) |
| --- | --- | --- | --- | --- |
| *algD* | algD-F (61) |  | CGTCTGCCGCGAGATCGGCT | 313 |
|  | algD-R (61) |  | GACCTCGACGGTCTTGCGGA |  |
| *lasB* | lasB-F (61) |  | GGAATGAACGAAGCGTTCTCCGAC | 284 |
|  | lasB-R (61) |  | TTGGCGTCGACGAACACCTCG |  |
| *toxA* | toxA-F (61) |  | CTGCGCGGGTCTATGTGCC | 270 |
|  | toxA-R (61) |  | GATGCTGGACGGGTCGAG |  |
| *plcH* | plcH-F (61) |  | GCACGTGGTCATCCTGATGC | 608 |
|  | plcH-R (61) |  | TCCGTAGGCGTCGACGTAC |  |
| *plcN* | plcN-F (61) |  | TCCGTTATCGCAACCAGCCCTACG | 481 |
|  | plcN-R (61) |  | TCGCTGTCGAGCAGGTCGAAC |  |
| *exoS* | exoS-F (61) |  | CGTCGTGTTCAAGCAGATGGTGCTG | 444 |
|  | exoS-R (61) |  | CCGAACCGCTTCACCAGGC |  |
| *rpsl* | rpsl-F (16) |  | GCAAGCGCATGGTCGACAAGA | 123 |
|  | rpsl-R (16) |  | CGCTGTGCTCTTGCAGGTTGTGA |  |
