# Supplementary material for: Prevalence of different virulence factors and their association with antimicrobial resistance among Pseudomonas aeruginosa clinical isolates from Egypt
Source: BMC Microbiol. 2023 Jun 3;23:161. doi: 10.1186/s12866-023-02897-8 (PMC10239191; doi:10.1186/s12866-023-02897-8)
Supplement: Supplementary file 6 — Additional file 6. PCR amplification conditions for genes encoding selected virulence factors. [file 12866_2023_2897_MOESM6_ESM.docx]

**Additional file 6:** PCR amplification conditions for genes encoding selected virulence factors.

| Virulence factor encoding gene | Thermal cycling conditions | | | | | Master mix used |
| --- | --- | --- | --- | --- | --- | --- |
|  | **Initial denaturation** | **30 cycles** | | | **Final extension** |  |
|  |  | **Denaturation** | **Annealing** | **Extension** |  |  |
| *algD* (53) | 94 °C/5 min | 94 °C/40 sec | 60 °C/1 min | 72 °C/2 min | 72 °C/10 min | OnePCR^TM^ |
| *lasB* (53) |  |  |  |  |  |  |
| *exoS* (53) |  |  | 63 °C/1 min |  |  |  |
| *plcH* (53) | 95 °C/1 min | 95 °C/15 sec | 60 °C/15 sec | 72 °C/10 sec | 72 °C/10 min | MyTaq^TM^ HS Mix |
| *plcN* (53) |  |  |  |  |  |  |
| *toxA* (53) |  |  |  |  |  |  |
